# The Epithelial Sodium Channel and the Processes of Wound Healing

**DOI:** 10.1155/2016/5675047

**Published:** 2016-07-14

**Authors:** Silvia Chifflet, Julio A. Hernandez

**Affiliations:** ^1^Departamento de Bioquímica, Facultad de Medicina, Universidad de la República, Gral. Flores 2125, 11800 Montevideo, Uruguay; ^2^Sección Biofísica, Facultad de Ciencias, Universidad de la República, Iguá 4225 esq. Mataojo, 11400 Montevideo, Uruguay

## Abstract

The epithelial sodium channel (ENaC) mediates passive sodium transport across the apical membranes of sodium absorbing epithelia, like the distal nephron, the intestine, and the lung airways. Additionally, the channel has been involved in the transduction of mechanical stimuli, such as hydrostatic pressure, membrane stretch, and shear stress from fluid flow. Thus, in vascular endothelium, it participates in the control of the vascular tone via its activity both as a sodium channel and as a shear stress transducer. Rather recently, ENaC has been shown to participate in the processes of wound healing, a role that may also involve its activities as sodium transporter and as mechanotransducer. Its presence as the sole channel mediating sodium transport in many tissues and the diversity of its functions probably underlie the complexity of its regulation. This brief review describes some aspects of ENaC regulation, comments on evidence about ENaC participation in wound healing, and suggests possible regulatory mechanisms involved in this participation.

## 1. Introduction

The capacity to respond to different types of injuries by healing processes is a major achievement of biological evolution and represents a crucial property for the homeostasis of an organism. Tissue restitution is a complex physiological response triggered by an injury. In the whole organism it is accomplished by the coordinated action of many cell types and involves distinctive sequential phases, that is, hemostasis, inflammation, proliferation, and remodeling [1, 2]. However, tissue culture studies have shown that isolated tissues already have the ability to restitute their integrity and have thus been proven to be a valuable tool in the study of wound healing. The healing responses are also activated during diverse pathological situations in the absence of an external injuring aggression, such as inflammatory bowel disease [3], gastric ulcers [4], asthma [5], tissue fibrosis [6, 7], and cancer [7, 8].

The epithelial sodium channel (ENaC) has been primarily considered to be a major participant in sodium and water homeostasis [9, 10]. This view was supported by its predominant presence in the distal nephron and other salt transporting epithelia. However, the channel can be found in a wide variety of cellular types and tissues [11] and has been suggested to participate in diverse other physiological processes, such as chemo- and mechanosensation [12], control of vascular tone [13–17], and sodium sensing and membrane potential regulation in the nervous system [18]. It has also been found that ENaC has a role in the processes of wound healing. The main objective of this work is to review evidence about the participation of ENaC in these processes. Prior to this, we briefly describe the general functional, structural, and regulatory characteristics of this channel with emphasis on aspects of interest for this review.

## 2. The Epithelial Sodium Channel

### 2.1. Physiological Roles

ENaC is a constitutively active, non-voltage-gated sodium channel typically located in the apical membranes of epithelial cells and is highly sensitive to inhibition by amiloride [11, 19, 20]. It is predominantly selective for sodium ions and exhibits a low single-channel conductance [21]. In salt-absorbing epithelia, ENaC mediates sodium entry in favor of its electrochemical gradient. In this type of cells, the sodium pump is fundamentally localized at the basolateral membrane, where it performs active extrusion of sodium. The sequential localization of the two transport systems along the apical to basolateral axis of the cell determines transepithelial flow of sodium ions, which is coupled to an equivalent flow of an accompanying monovalent anion, most commonly chloride [22]. The overall process is therefore pumped by the sodium-potassium ATPase. This basic scheme accounts for sodium absorption in many epithelia, especially the distal nephron, the colon, and the lungs [19], where the channel participates in fundamental physiological functions. In this way, aldosterone-regulated sodium reabsorption in the kidney plays a major role in the control of extracellular fluid volume, blood pressure, and ionic renal secretion [23, 24]. Several inherited diseases involving alterations in sodium homeostasis, such as Liddle's syndrome and pseudohypoaldosteronism type I, are a consequence of ENaC mutations [11, 25]. The above-described scheme also accounts for sodium absorption in the colon [22]. In the lungs, the absorption of sodium contributes to the maintenance of the normal composition of the surface liquid in the airways [26, 27]. ENaC has been identified in other epithelial tissues, such as vascular endothelia, where it contributes to the maintenance of the vascular tone [13–16]. It is present in corneal endothelium [28], where it plays a fundamental role in salt and fluid transport necessary for the maintenance of the organ transparency [29]. Besides its role in sodium metabolism, ENaC has also been proposed to participate in the transduction of varied mechanical stimuli, such as shear stress, hydrostatic pressure, and membrane stretch [30]. It is interesting to note that ENaC seems to be the only channel mediating apical sodium transport in some tissues, like colon [31], cortical collecting duct of the kidney [24], and airway epithelia [32]. Together with its physiological importance, this characteristic may explain the very complex regulatory mechanisms controlling the channel activity, as commented in the following sections.

### 2.2. Basic Structural Aspects and Interactions

ENaC is a member of the ENaC/degenerin superfamily, which also includes the acid-sensing ion channels (ASICs), the pickpocket proteins found in* Drosophila* and other Diptera, and the nematode degenerins. It is an heterotrimer composed of three homologous subunits, *α* (or *δ*), *β*, and *γ*. For details concerning distribution, gene coding, and protein structure of ENaC, the reader can consult several recent comprehensive reviews [11, 25, 33]. Of interest to this work is the fact that each ENaC subunit possesses a very large extracellular domain, which is the site of many regulatory effects (see below). The intracellular domains correspond to the carboxyl and amino terminals of each subunit. At these levels, it has been suggested that ENaC interacts with a multiprotein regulatory complex that includes, among others, the ubiquitin protein ligase Nedd4 (neuronal precursor cell-expressed developmentally downregulated 4) and the serum and glucocorticoid-regulated kinase 1 (SGK1) [34]. Besides these regulatory sites, at the intracellular level, ENaC establishes diverse interactions with the cytoskeleton. Its localization at the apical membrane is maintained by its direct binding to spectrin [35] and ankyrin [36]. ENaC also binds to the actin cytoskeleton, either directly [37] or through intermediary proteins [38–40]. There is evidence that these bindings of ENaC to the actin cytoskeleton modify the channel conductance [38, 40–42]. Moreover, the interactions of ENaC with the cytoskeleton mediate some of its mechanosensitive responses [43].

### 2.3. ENaC Regulation

**Figure 1:**
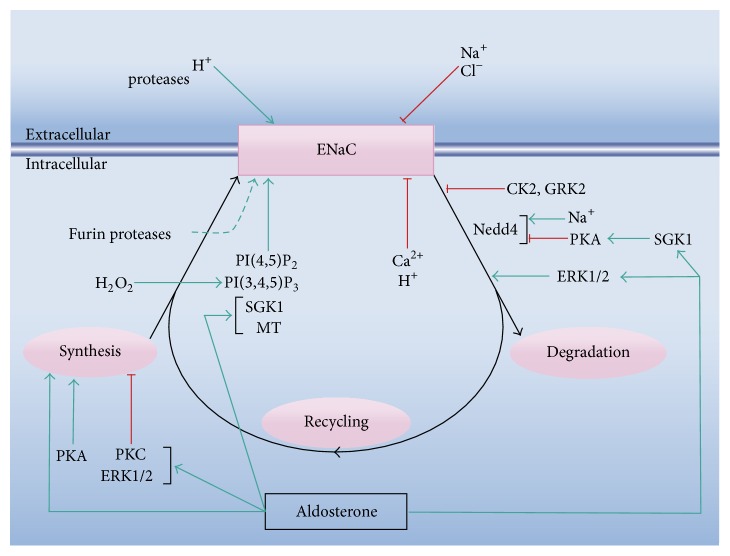
Scheme of the basic mechanisms of ENaC regulation. The scheme indicates the effects of several modulators on the channel activity and/or expression. The three levels of regulation of ENaC are integrated in the scheme: (i) modulation of the kinetic properties of the channel in situ at the plasma membrane or at its trafficking, (ii) regulation of its synthesis and retrieval from the plasma membrane, and (iii) the effects of first and second messengers on the signaling networks that modulate the channel's activity, with emphasis on aldosterone. MT: methyltransferases. See the main text for a detailed description of the regulatory effects.

ENaC is subject to a complex regulation that involves a myriad of mechanisms, summarized in Figure 1. The reader is referred to more detailed reviews on this topic [30, 34, 44–46]. The regulation of ENaC activity can be schematically considered to occur at three levels: (i) by modulation of the kinetic properties of the channel (i.e., modifying the open probability or the single-channel conductance), (ii) by regulation of its amount at the plasma membrane, and (iii) by the effects of first and second messengers on the signaling networks that modulate the channel's activity. The latter two levels include regulatory effects at its synthesis, storage, trafficking to the plasma membrane domains, and membrane insertion and retrieval. In essence, the complexity of ENaC regulation emerges from the fact that the three levels are strongly interrelated (Figure 1). As an example, it has been proposed that SGK1 increases ENaC activity by direct phosphorylation [47], by phosphorylation of Nedd4-2 [48–50], and by augmenting the transcription of the *α* subunit [51]. In turn, SGK1 is at the crossroad of different signaling pathways, like those triggered by aldosterone, insulin, IGF-1, and mTOR [46, 52].

The channel can be directly inhibited at the extracellular domains by sodium [53–56] and chloride [57, 58] and activated by acidic pH [59]. At this level, several proteases affect the open probability of the channel [30, 32, 44]. On the intracellular side, the channel is inhibited by calcium [60] and acidic pH [60–62] and activated by phosphatidylinositol(3,4,5)-trisphosphate (PI(3,4,5)P_3_) and phosphatidylinositol (4,5)-bisphosphate (PI(4,5)P_2_) [63–67]. H_2_O_2_ determines a rise in PI(3,4,5)P_3_ and thus produces ENaC stimulation [68]. Intracellular sodium determines inhibition of epithelial ENaC, an effect known as feedback inhibition [69, 70] which is mediated by sodium interference with ENaC proteases [71, 72].

At the plasma membrane, the channel molecules can be both in mature (active) or immature (inactive) forms, depending on their posttranslational processing [73]. ENaC is synthesized in inactive form and is activated by limited proteolysis of the extracellular domains [74]. ENaC can be activated during its trafficking to the membrane by the trans-Golgi serine protease furin and inserted already in active form [73, 75].

The retrieval of ENaC involves, among others, the ubiquitin ligase Nedd4 [34, 76]. Phosphorylation of Nedd4 by SGK1 inhibits ENaC ubiquitination and thus determines an increase in the channel activity [45]. CK2 (casein kinase 2) and GRK2 (G protein-coupled receptor kinase 2) stimulate ENaC activity by direct phosphorylation at sites that interfere with Nedd4, while other kinases (e.g., the extracellular signal-regulated kinases 1 and 2, ERK1/2) perform phosphorylation of channel sites that favor Nedd4 interaction (ibid). In addition, intracellular sodium stimulates Nedd4 [77]. After internalization, the ENaC molecules can either be recycled or be degraded. Besides aldosterone, the main regulator of ENaC synthesis (see below), other effectors promote or inhibit the channel transcription. For instance, PKA (protein kinase A) has been suggested to activate the transcription of *α*-ENaC, while PKC (protein kinase C) and ERK1/2 seem to inhibit the transcription of the three subunits and of the *α* subunit, respectively [45].

In the whole organism, ENaC activity is subject to the control of several hormones and growth factors [25], most significantly aldosterone, vasopressin, and insulin. For the regulation of ENaC activity, aldosterone behaves both as a transcriptional and as a nontranscriptional effector (for detailed reviews, see [24, 25, 78]). Briefly, aldosterone binds to the cytoplasmic mineralocorticoid receptor (MCR) and translocates to the nucleus, where, among other target genes, it promotes transcription of ENaC subunits in a tissue-specific manner [24, 79]. It also stimulates transcription of SGK1 [45, 46]. It is to be noted that binding to the MCR also stimulates the epidermal growth factor receptor that, in turn, prevents excessive activation of ENaC via ERK signaling [80, 81]. Aldosterone possesses several nongenomic effects affecting ENaC activity, such as activation of ERK1/2 and p38, activation of members of the PKC family, and stimulation of cAMP and cytosolic calcium increases [82]. Other nongenomic effects include methylation of *β* ENaC [83–85] and vascular endothelial cell swelling [86]. From the above, aldosterone can be considered as a crucial element in the crosstalk between regulatory pathways of ENaC activity.

The majority of the regulatory properties of ENaC summarized in Figure 1 were described for the distal nephron and other salt-absorbing epithelia. There are significant regulatory differences, however, with other noncanonical localizations, such as vascular endothelium, where the transtissular transport of sodium does not represent the main physiological role of the channel [16].

### 2.4. ENaC Participation in Mechanotransduction

The above-summarized regulatory mechanisms were mostly concerned with the role of ENaC in sodium metabolism. The channel also participates in the transduction of mechanical stimuli. Evidence for ENaC activation by mechanical stimuli has been reported since the late eighties [93]. Three main types of these stimuli are transduced by ENaC: hydrostatic pressure, membrane stretch, and shear stress of fluid flow. The role of the channel in the transduction of hydrostatic pressure differences and modifications in membrane stretch has been demonstrated both in* Xenopus* oocyte expression experiments [94–96] and in native epithelia [97, 98]. In diverse epithelia lining tubular structures subject to fluid flow, such as renal epithelia and vascular endothelium, ENaC mediates the transduction of shear stress [17, 99–102]. As mentioned, the cellular responses to hydrostatic pressure differences and to modifications in membrane stretch, mediated by ENaC, depend upon interactions with the cytoskeleton [43]. However, activation of ENaC by shear stress does not seem to depend on the interaction of the channel with the actin cytoskeleton [103].

## 3. ENaC and Wound Healing

### 3.1. General Aspects of the Healing Processes

**Figure 2:**
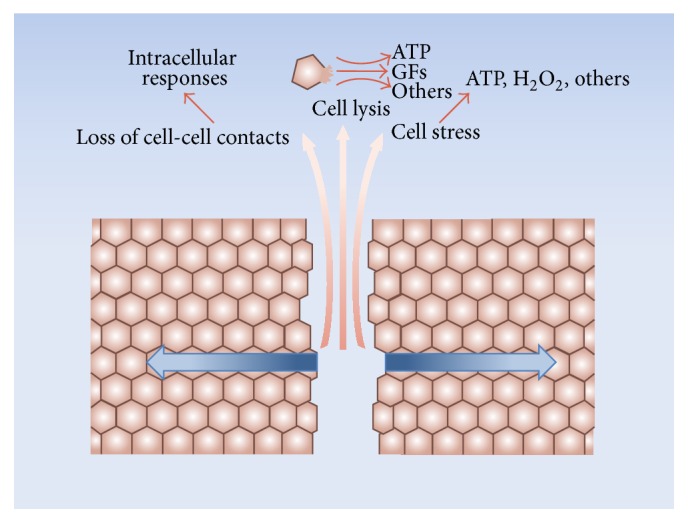
Initial events after a mechanical injury on an epithelial monolayer under culture conditions. A mechanical scratch determines three main initial events: (i) death and lysis of the injured cells, with release of cellular content, (ii) development of mechanical stress, and (iii) loss of cell-cell contacts of the remaining living cells at the wound borders. Among the intracellular molecules released, some that play a role in wound healing in in vitro conditions could be ATP [87, 88] and growth factors (GFs) [89, 90]. ATP, H_2_O_2_, and eicosanoids are released by the surviving border cells in response to stress [87, 88]. The loss of cell-cell contacts at the free wound edges stimulates migration and activates growth factor receptors [91, 92]. The three types of events trigger different responses of the surviving cells, frequently propagated to the rest of the monolayer as physical and/or chemical gradients (blue arrows).

Confluent cultures of epithelia and other cellular types are useful experimental models to study the basic aspects of the healing responses to wounds produced by mechanical or chemical agents. Most of our present knowledge regarding tissue restitution has been obtained from this type of studies. Even under these simple in vitro conditions, the healing response is complex and involves several aspects. For the case of a mechanical scratch on an epithelial monolayer, three main initial events can be schematically described: (i) death and lysis of injured cells, with release of cellular content, (ii) development of mechanical stress, and (iii) loss of cell-cell contacts of the remaining living cells at the wound borders (Figure 2). All these events trigger different signaling pathways of the surviving cells. Since the signals are generated at the site of the wound, there will be a signaling gradient from the edge of the injury towards the undamaged monolayer, providing, among others, positional information to the healing cells. Some of the responses of the surviving cells that develop early after wounding are the fast calcium wave [104–112], the hydrogen peroxide gradient [112, 113], and the ERK1/2 waves [114]. In turn, these responses trigger signaling cascades in the healing cells.

**Figure 3 fig3:**
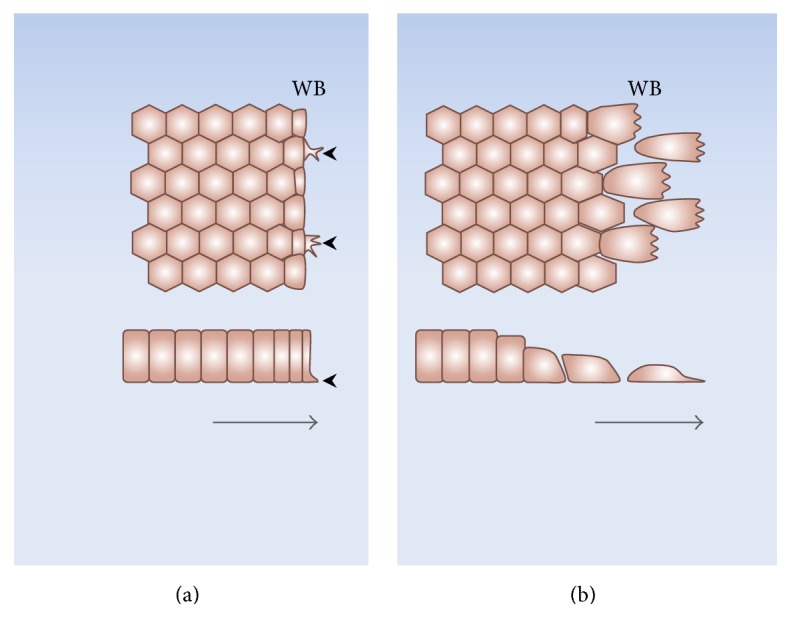
Morphological cellular changes during wound healing of cultured epithelia. In the actin-cable mode (a) the healing cells mainly migrate in compact collective fashion and conserve the epithelial phenotype to a greater extent. Some cells at the wound border develop small lamellipodia (arrowheads) that drag the tissue towards the injured area. In the lamellipodial mode (b) the border cells progressively lose their apicobasal polarity and the cell-cell contacts and acquire well-developed lamellipodia. The lateral views (lower panels) show that while in the actin-cable mode the border cells conserve their morphology, in the lamellipodial mode the cells at the wound edge flatten and detach from their neighbors. WB: wound border. The arrows indicate the direction of healing.

Later in the healing process, the border cells progressively undergo dramatic morphological changes (Figure 3). The modifications are more conspicuous at the cells of the wound borders, but the neighboring cells also undergo changes up to several rows from the border. In essence, the cells modify their shapes and start to migrate into the denuded area, either individually, losing their cell-cell junctions, or as a cohesive sheet, maintaining these junctions. Besides migration, the other relevant cellular process triggered to cover the injury zone is proliferation. The extent to which proliferation contributes to wound healing depends on the particular cell type. For the case of bovine corneal endothelial (BCE) cells in culture, DNA synthesis at the leading edge starts to increase 8–10 hours after injury [115], whereas for calf pulmonary artery endothelial cells it begins at 18–24 hours [116]. The morphological changes of the healing cells depend upon the modality of wound closure. Thus, in the actin-cable mode, the cells migrate in a predominantly compact collective fashion and conserve the typical epithelial phenotype to a greater extent (Figure 3(a)), whereas in lamellipodial crawling the cells lose their apicobasal polarity and cell-cell contacts, undergoing more conspicuous morphological changes (Figure 3(b)) [117].

Another important characteristic of the processes of wound healing is the development of electric fields between the injured and uninjured zones. These electric fields represent major cues to stimulate and establish the direction of cell migration during tissue repair [118, 119]. The electrical phenomena observed during wound healing are analogous and have similar significance to those that take place during individual or collective cell migration in morphogenesis [118, 120].

In essence, wound healing is a complex process involving diverse cellular modifications, among which migration plays a leading role. The functional and morphological changes experienced by migrating cells are diverse and have been thoroughly investigated. In this respect, ionic transport has been recognized as a key player of this process [121, 122].

### 3.2. ENaC and Wound Healing

In recent years, evidence has emerged reporting that ENaC activation is relevant for the healing process in several cellular types. Thus, an increase in ENaC expression occurs during wound restitution in monolayers of BCE cells in culture which determines plasma membrane depolarization and increase in cytosolic sodium of the healing cells, whose inhibition significantly reduces the velocity of healing [123]. The participation of ENaC in these processes has also been proposed from studies in other cellular systems, both in culture [124–127] and in situ [128].

Work from Drummond's laboratory [124] demonstrated that vascular smooth muscle cells express ENaC and that pharmacological inhibition of the channel or silencing of its gene expression determines a decrease in the rate of wound healing. In another set of experiments, authors from this group reported that *β*-ENaC mediates cytotrophoblast migration and that the increase of its expression enhances individual cell migration [129]. In BeWo cells, a line of human hormone-synthesizing trophoblastic cells, Del Mónaco and coworkers [125] found that an increase in the immunofluorescence signals of the three ENaC subunits takes place during wound healing. They also observed that, in these cells, aldosterone provokes an amiloride-inhibitable rise in the velocity of healing and an augmented expression of *α*-ENaC. More recent work in BeWo cells from this laboratory shows that the aldosterone effect on the healing rate is at least partially due to an increase in ENaC activity by methylation [130]. From studies on a line of human keratinocytes, Yang and coworkers [126] could establish that ENaC is crucial for directional migration in an electric field by allowing stabilization of the lamella at the cathodal cellular side. Also, that electrically induced directional migration is blocked by pharmacological inhibition or genetic silencing of the channel. The importance of these results for the processes of wound healing rests on the finding that, as commented, electric fields spontaneously develop during these processes. Further work from our laboratory showed that, besides BCE, other epithelia in culture (e.g., rabbit corneal epithelia and Madin-Darby canine kidney (MDCK) cells) also exhibit an increase in ENaC expression and the consequent ionic and electrical modifications during tissue repair [127]. However, this work additionally showed that such changes do not take place in bovine aortic endothelial cells (BAEC). Nevertheless, the study also provided evidence that, in BAEC, pharmacological inhibition of ENaC decreases the velocity of healing, while aldosterone and forskolin increase it.

As a precedent to these studies, Rajnicek and coworkers [131] found that an amiloride-sensitive endogenous sodium current was critical for* Xenopus* neurula wound healing. More recently, the effect of amiloride on the healing processes has been observed in other systems. Thus, amiloride inhibits reepithelization of migrating epidermal tongue in situ and in culture [132]. In both mouse and human skin, application of the drug produced significant decreases in the endogenous electrical fields generated by wounds [133]. Amiloride is a widely employed diuretic agent with negligible side effects [134]. This is possibly the reason for the lack of studies on the effects of the drug on wound healing in human patients. More direct evidence on the role of ENaC in wound healing in whole organisms is scarce, mostly due to the fact that ENaC knockout mice develop respiratory stress and die shortly after birth [126, 135, 136].

In BCE cells in culture, the ENaC-mediated effects on cellular sodium concentration and membrane potential start to become evident 1-2 hours after wounding and progressively extend to the unwounded monolayer [123]. Concomitant with these phenomena, we could observe the development of a sustained cytosolic calcium rise that temporarily and spatially overlaps with the sodium increase [111]. In this study we also showed that this slow calcium wave (SCW) results from the functional coupling between ENaC and the sodium calcium exchanger (NCX) working in the reverse mode. Also, that blockade of the calcium increase by pharmacological inhibition of NCX reduces the velocity of healing. As described (Figure 1), an elevation in cytosolic calcium constitutes an inhibitory signal of ENaC activity. However, in epithelia that develop a SCW during wound healing, this sustained calcium rise does not prevent the increase in ENaC activity [111].

Besides its role in wound healing, ENaC has been demonstrated to participate in individual cell migration of normal [137, 138] and malignant [139, 140] cells.

### 3.3. Possible Signals Modifying the Expression and/or Activity of ENaC during Wound Healing

**Figure 4:**
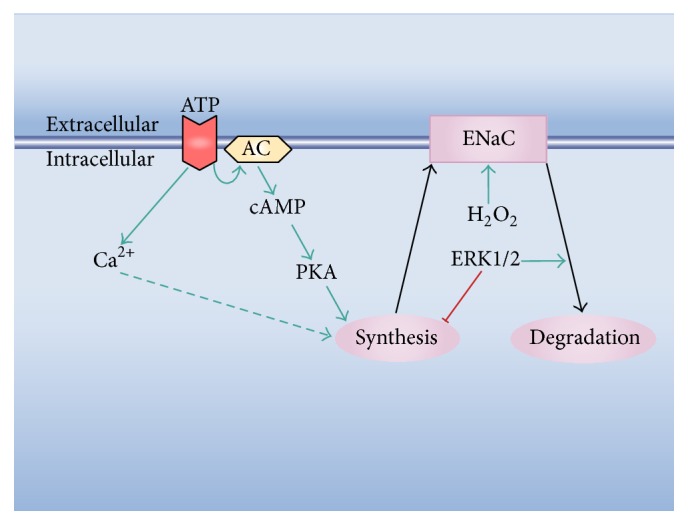
Possible signaling pathways involved in ENaC activation during wound healing. The scheme highlights possible effects of some signals triggered early in the healing process, that is, the calcium, H_2_O_2_, and ERK1/2 waves (cf. Figure 1). The dashed arrow indicates that the suggested calcium effect does not take place in some cell types. ATP binds to its plasma membrane receptor and determines an increase in cytosolic calcium. For the case of the P2Y_11_ receptor, ATP binding also stimulates the adenylyl cyclase (AC).

As described above, there is evidence that, in some epithelia, ENaC increases both its expression and activity during wound healing. This process is noticeable a few hours after injury and progressively evolves from the wound border cells towards the rest of the monolayer. The specific signals responsible for these increases are not yet known. In the whole organism, ENaC is subject to regulation by diverse hormones and growth factors, as summarized in previous sections. However, under tissue culture conditions, the stimuli to modify ENaC expression during wound healing are restricted to those resulting from the injured tissue (Figure 2 [Fig fig3]). As mentioned, these include release of the content of the damaged cells, loss of cell-cell contacts of the edge cells, and mechanical stress of the border and neighboring cells. These stimuli in turn trigger many signaling cascades. The calcium, hydrogen peroxide, and ERK1/2 waves are among the earliest signals detected in response to the injury (see above) and could be involved in the modification of the channel expression and/or activity (Figure 4). Besides other possible regulatory effects, the fast calcium wave has been proposed to activate immediate early genes [106] and thus constitutes a good candidate for stimulation of ENaC expression. However, for the case of BCE cells, we found that the reversible inhibition of this wave fails to block the increase in the channel expression observed during wound healing [111]. To date, there is no evidence regarding whether any of the other two waves participates in the response. Hydrogen peroxide activates ENaC in its plasma membrane location (Figure 1). However, there are no reports suggesting a possible role of H_2_O_2_ in the channel synthesis. ERK1/2 inhibits ENaC synthesis and stimulates its retrieval from the plasma membrane (Figure 1). As commented above, this could represent a mechanism of feedback inhibition to prevent an excessive increase in the channel expression [81]. Alternatively, diverse molecules released from the damaged cells could per se represent stimuli to enhance ENaC expression (Figure 2). Among these molecules, ATP was found to be responsible for the generation of the fast calcium wave [105, 108, 141–145]. Besides this role, ATP could also trigger other signaling pathways. For instance, its binding to the P2Y_11_ receptor activates adenylyl cyclase [146, 147], thus increasing cAMP and consequently activating PKA. In turn, PKA stimulates ENaC synthesis (Figure 1).

Although the increase in ENaC expression could be the sole responsible for the gradual rise in its activity during wound healing in several epithelia, other additional stimuli could be involved. A potential candidate for this role is the H_2_O_2_ wave. As mentioned, H_2_O_2_ can stimulate the channel via a phosphatidylinositide 3-kinase-dependent pathway [68] (Figure 1). ENaC activation can also be produced by actin. In this respect, it has been shown that small fragments of F-actin stimulate the channel [37, 41, 148]. Hydrogen peroxide activates cofilin, which in turn promotes actin filament severing and depolymerization [149]. In this way, H_2_O_2_ could further participate in the regulation of ENaC activity via the indirect generation of small actin fragments. In addition, modifications in the cytoskeleton produced as a consequence of the mechanical stress of the border cells (Figure 2) could also modify the channel activity. In this respect, the close interactions established between the cytoskeleton and ENaC mediate bidirectional functional effects, where the channel is capable of modifying the cytoskeletal organization via the transduction of mechanical signals and the cytoskeleton affects its activity by direct structural interactions (see above). Other mechanisms have been proposed for in situ ENaC stimulation. For instance, in injured skin, the increase in extracellular sodium activates ENaC via the sodium sensing channel Na_*x*_ [150].

### 3.4. Possible Mechanisms Mediating the Effects of ENaC on Wound Healing

**Figure 5:**
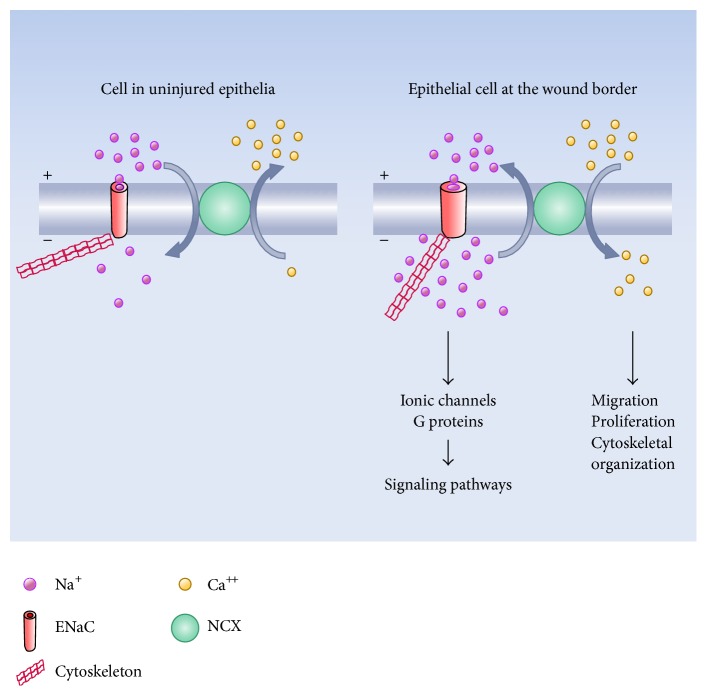
Possible effects of ENaC activation on the healing process. The scheme summarizes some effects of the increase in ENaC expression and/or activity. The direct consequences of the channel stimulation are an elevation in intracellular sodium and plasma membrane depolarization (represented by smaller plus and minus symbols). The scheme also suggests a possible effect on the cytoskeletal organization provoked by the ionic and electrical changes. In a secondary fashion, the rise in intracellular sodium and plasma membrane depolarization determine the reverse mode of operation of the sodium-calcium exchanger (NCX) with the consequent rise in cytosolic calcium. The sodium and calcium increases constitute signals that modulate diverse cellular processes.

In principle, ENaC participation in the processes of wound healing can be considered to be mediated by two types of mechanisms (Figure 5): (i) those dependent on the ionic and electrical consequences of the increased channel activity, that is, increase in intracellular sodium and plasma membrane depolarization, and (ii) those dependent upon the role of the channel as a mechanotransducer and/or as an intermediate of signaling pathways regulating cytoskeletal organization.

The ENaC-mediated increase in cellular sodium and plasma membrane depolarization of the healing cells could per se represent signals to trigger cellular modifications. For instance, increases in intracellular sodium have signaling roles in smooth muscle cells [151] and astrocytes [152, 153]. Among other possible pathways, sodium has been shown to modify the interaction between *α* and *βγ* subunits of G proteins, thus modifying ionic channel activity [154, 155] and potentially affecting other G protein-dependent signaling pathways [156]. For the case of the healing processes, intracellular sodium increase and concomitant membrane depolarization mediated by the voltage-gated Na_V_1.2 sodium channel play a fundamental role in tail regeneration in* Xenopus laevis* tadpoles [157].

As commented above, during wound healing in BCE monolayers, there is an elevation in cytosolic calcium as a consequence of sodium increase and membrane depolarization, due to the fact that these conditions promote the reverse functioning of the NCX [111]. The rise in cytosolic calcium of these cells could constitute a signal to trigger diverse cellular modifications [158], in particular migration [121, 122, 159–162] and proliferation [163]. An analogous mechanism involving sodium rise and secondary calcium increase due to the coupling between ENaC and the NCX functioning in reverse mode has been suggested to promote migration in NG2 cells, a line of oligodendrocyte progenitor cells [164], microglia [165], cardiac myofibroblasts [166], tendon fibroblasts [167], and pancreatic cancer cells [168]. Besides its effect on plasma membrane NCX, cytosolic sodium increase also determines reverse functioning of the mitochondrial NCX [169] and mobilization of calcium from the endoplasmic reticulum [170].

It has been proposed that changes in the plasma membrane potential could participate in diverse cellular signaling pathways [171–173]. As a contribution to this concept, we were able to establish that, in BCE and other epithelial cells in confluence, plasma membrane depolarization determines a cytoskeletal reorganization characterized by a gradual loss of the peripheral actin ring, an increase in the amount of F-actin throughout the cytoplasm, and appearance of intercellular gaps with eventual cell detachment [174, 175]. Cell migration involves dramatic modifications in the cytoskeletal organization. It is therefore possible that the cytoskeletal changes of the healing cells are partly determined by the plasma membrane depolarization. Evidence from our studies suggests that the increase in intracellular sodium could also have a role per se in the cytoskeletal reorganization [123].

The interactions between ENaC and the cytoskeleton mediate some of the responses of the channel to mechanical stimuli [43]. For the case of wound healing, physical injuries generate, among others, traction and pressure forces on the surviving cells (Figure 2) that could be transduced by cellular paths involving ENaC. The connections of the channel with the cytoskeleton can also mediate the propagation of electrical signals [176]. This property could participate in the reorganization of actin provoked by membrane depolarization (Figure 5).

### 3.5. Physiological Significance of ENaC Participation in Wound Healing

As described in the previous sections, there is evidence suggesting that ENaC is an important player in the processes of wound healing. Several characteristics of the channel may underlie this role:

1. ENaC constitutes the main, and in some epithelia the only, pathway for sodium entrance to the cells. Thus, the modulation of its activity represents the fundamental mechanism of regulation of the intracellular sodium content.
2. The increases in ENaC expression and/or activity determine several simultaneous cellular effects: rise in intracellular sodium concentration, plasma membrane depolarization, and modifications in cytoskeletal organization. In turn, the elevation in sodium and the plasma membrane depolarization produce secondary ionic effects, like calcium increase.
3. Its intricate regulation ensures a precise control of its activity.
4. The ionic modifications (e.g., in sodium and calcium) produced by an increment in ENaC activity constitute signals for many cellular events.
5. The macroscopic electric fields that could emerge as a consequence of ENaC-dependent membrane depolarization during wound healing could participate in the processes of orientation of cell migration.

In summary, the modulation of a single transport system may affect several cellular properties of importance for wound healing. In the whole organism, besides the signals that could modulate ENaC expression in vitro, other effectors could be involved, such as cytokines and growth factors. Further research on the role of ENaC in tissue restitution is certainly needed, both in vitro and, particularly, in the whole organism. This should include elucidation of the signals affecting its regulation, the mechanisms involved in its participation in the healing process, and its importance in diseases, where these mechanisms are affected.

## 4. Conclusions

The epithelial sodium channel (ENaC) mediates passive sodium entry across the apical membranes of transport epithelia. In kidney, intestine, and lungs this function is of critical physiological importance for the whole organism. ENaC is also involved in the transduction of mechanical stimuli, a property relevant to its participation in the control of the arterial tone. Besides these localizations, ENaC has been identified in many other epithelial and nonepithelial cell types, where its physiological roles may depend on its properties both as sodium channel and as mechanotransducer. ENaC is subject to a very complex regulatory scheme, possibly as a consequence of its physiological relevance, its presence in many cell types, and the fact that it may be the only channel mediating apical sodium transport in some epithelia.

Recent findings reveal an important contribution of ENaC to the processes of wound healing. The evidence suggests that this participation involves both its activity as a sodium channel and as a mediator of mechanotransduction. An increase in ENaC expression occurs in the edge cells of several epithelial cells in culture in the course of tissue restitution. This increase and/or regulatory effects on immature channels are the causes of the sustained rise in cellular sodium and plasma membrane depolarization observed in these cells. Pharmacological inhibition of the sodium channel or blockage of its synthesis significantly reduces the velocity of healing. The modifications in cellular sodium and in the membrane potential in turn determine secondary responses. Thus, cytosolic calcium of the healing cells, a fundamental second messenger of many processes, gradually augments as a consequence of a functional coupling between ENaC and the sodium-calcium exchanger. The cytoskeletal reorganization that the migrating cells undergo in the course of wound healing has been at least partially considered to depend on membrane depolarization. Since these findings were obtained under in vitro conditions, the basic signals involved in the increase in ENaC expression and activation during tissue restitution are restricted to those resulting from the injured cells and include release of the content of the damaged cells, loss of cell-cell contacts of the edge cells, and mechanical stress of the border and neighboring cells. At present, the nature of these signals is unknown and is the object of current investigation.
